# Effect of mouthwashes on the microhardness of aesthetic composite restorative materials

**DOI:** 10.23938/ASSN.1049

**Published:** 2023-08-30

**Authors:** Noura Abdulaziz Alessa

**Affiliations:** King Saud University Department of Pediatric Dentistry and Orthodontics College of Dentistry King Saud University Riyadh Saudi Arabia

**Keywords:** Resin composite, Vickers’ Hardness, Mouthwashes, Microhardness, pH, Composite, Dureza de Vickers, Colutorio bucal, Microdureza, pH

## Abstract

**Background:**

Mouthwashes are increasingly being used worldwide. However, these preparations are known to have a negative impact on composite resin dental restorations. In this study, we aim to evaluate the effect of mouthwashes on the microhardness of such restorations.

**Methods:**

Thirty specimens of Tetric N-Ceram composite were prepared. Each composite specimen was cured for 40 seconds and kept in saline solution for 24 hours at 37 °C. Baseline microhardness of each specimen was recorded using an Innovatest Vickers Micro Hardness Tester. Composite specimens were randomly placed in 20 mL of the selected mouthwashes (Colgate® Plax, Listerine® Teeth & Gum Defence, and Closeup® Antibacterial Mouthwash Cool Breeze) and stored in an incubator for 24 hours at 37 °C. Next, microhardness values were rechecked. pH measurements were recorded for each type of mouthwash using a digital pH meter.

**Results:**

Due to the acidic nature of Colgate® and Listerine®, the microhardness of the restorations decreased with these mouthwashes; Listerine® caused the greatest decrease in microhardness and had the lowest pH reading (4.34). For Closeup®, with a neutral pH (7.02), no negative effect on microhardness was found; on the contrary, due to the presence of zinc in this latter mouthwash, an increase of the microhardness was found.

**Conclusions:**

We confirm the negative effect of acidic mouthwashes on the microhardness of composite dental restorations.

## RESUMEN

**Fundamento:**

El uso de colutorios bucales está aumentando a nivel mundial. Sin embargo, es conocido que afectan negativamente a las reparaciones dentales de composite, por lo que el objetivo de este estudio es evaluar el efecto de los colutorios bucales sobre la microdureza de estas reparaciones.

**Métodos:**

Se prepararon treinta muestras de composite Tetric N-Ceram. Cada muestra compuesta se curó durante 40 segundos y se mantuvo en solución salina durante 24 horas a 37 °C. La microdureza inicial de cada muestra se registró utilizando un probador de microdureza Innovatest Vickers. Las muestras se sumergieron aleatoriamente en 20 mL de los colutorios bucales seleccionados (Colgate® *Plax,* Listerine® *Teeth & Gum Defence* y Closeup® *Antibacterial Mouthwash Cool Breeze*) y se incubaron a 37 °C durante 24 horas. A continuación, se volvieron a comprobar los valores de microdureza. El pH de cada colutorio bucal empleado se midió con un pHmetro digital.

**Resultados:**

Debido a la naturaleza ácida de Colgate® y Listerine®, la microdureza de las muestras de composite sumergidas en estos colutorios disminuyó; Listerine® causó la mayor disminución de microdureza y mostró el pH más bajo (4,34). Closeup®, con pH neutro (7,02), no mostró ningún efecto negativo sobre la microdureza; por el contrario, su contenido en zinc aumentó la microdureza de las muestras.

**Conclusiones:**

Los colutorios bucales ácidos mostraron un efecto negativo sobre la microdureza de las restauraciones dentales de composite.

## INTRODUCTION

Tooth-coloured restorations are currently in high demand as they mimic the natural appearance of the teeth. Composite resins have therefore become important dental restorative materials due to their aesthetic features, as well as improved physical and mechanical properties; furthermore, it is a minimally invasive dental procedure^1^^,^^2^. Drinks, food, and the oral environment, including saliva and pH, affect the mechanical properties of dental resins^3^^,^^4^, which may cause changes in the wear resistance of the resin composite and a reduction in surface hardness^5^. Consequently, there is increased surface roughness, leading to unfavourable plaque accumulation, staining of the composite, and eventual failure of the restoration, thus impacting the longevity, durability, and degradation of the restorations^6^^,^^7^.

Mouthwashes are widely used worldwide^8^ to try to solve various dental problems even without a written prescription; they are used as adjuvants in treating gingivitis and periodontitis, halitosis, and to help prevent caries. There are many different types of mouthwashes, each with different components such as fluoride, antimicrobial agents, preservatives, salt, alcohol, and various flavors^9^. However, regular use of mouthwash may have a negative effect on dental restorations and/or oral tissues.

Frequent use of mouthwashes may increase the risk of pigmentation, dry mouth, and changes in the physical properties of composite resin restorations^10^. The extent of the changes of the physical properties of the composites, such as microhardness, mainly depends on the type and composition of the used materials^9^. Alcohol-containing mouthwashes cause wear and surface degeneration in dental restorations. Other materials present in mouthwashes, such as detergents, emulsifiers, and organic acids, may also have adverse effects on composite restorations^9^.

An ideal restorative material should function as natural teeth and be equally durable, which requires sufficient values of mechanical properties such as hardness, compressive strength, and flexural strength^11^^,^^12^. Composite restorations are mostly used in clinical practice to replace old dental restorations because of their aesthetics, ease of use, and lower invasiveness to the tooth structure^13^.

Several factors can affect the long-term durability of composite restorations including secondary caries or fractures^12^. The microhardness of restorative materials is an important feature, as it must be able to withstand intraoral compressive strength and be resistant to softening^14^; a low surface hardness is more prone to wear, resulting in fracture^15^.

Different types of mouthwashes are available in the market and many have not been studied to determine their effects on dental restorations. In this study we aimed to evaluate the effect of several commercial mouthwashes on the microhardness of aesthetic composite restorative materials.

## METHODS

This experimental study was developed and approved (Ref. FR0609) by the College of Dentistry Research Centre, at the King Saud University (Riyadh, Saudi Arabia).

### Composite restorations preparation

For this study, we selected Tetric N-Ceram composite (Ivoclar Vivadent Inc., Amherst, New York, USA). This composite contains about 19-20% of dimethacrylates, ytterbium trifluoride, barium glass, additives, initiators, stabilizers, pigments, and about 80% of mixed oxide and copolymers.

Thirty cylindrical composite specimens were prepared with a diameter of 10 mm and 3 mm height with the aid of a stainless-steel mould. Each mould was placed on a glass slide and filled with the resin composite to a slight excess using a composite filling instrument. Next, each specimen was covered with a clear matrix strip, and another glass slide was placed on the top to remove excess material and obtain smooth surfaces. Each specimen was cured for 40 seconds from the top and bottom using a LED light cure unit, following the instructions provided by the manufacturer. All specimens were kept in saline solution for 24 hours at 37 °C. The baseline microhardness of each specimen was recorded using an Innovatest Vickers Micro-Hardness Tester (Innovatest, New York, USA) with a load of 300 g and a dwell time of 15 s. Three points on each specimen with a distance of 1 mm between them were selected to obtain microhardness readings, and the averages of the three readings were calculated for each specimen.

### Immersion of composite specimens in mouthwashes

We selected three commercially available mouth-washes for this study: Closeup® Antibacterial Mouthwash Cool Breeze (Unilever, Russia), Colgate® Plax (Colgate-Palmolive, Thailand), and Listerine® Teeth & Gum Defence (Johnson & Johnson, Italy); the composition of each mouthwash is detailed in Appendix I.

The 30 composite specimens were randomly divided into three groups of ten specimens; each group corresponded to one of the selected mouthwashes. All specimens were immersed in 20 mL of the selected mouthwash for 24 h at 37 °C in an incubator, which almost equals 2 min of rinsing daily for two years; the specimens were then removed from the mouthwashes and dried under air at room temperature (25°C).

The surface microhardness was rechecked as described above for baseline records. The pH was measured for each mouthwash using a Mettler Toledo® SevenEasy pH digital pH-meter (Mettler-Toledo, Schwerzenbach, Switzerland).

### Statistical analysis

Microhardness values for each group were presented as mean and standard deviation (SD). Pre versus post immersion comparisons were carried out using Student’s paired t-test. Mean differences (pre- and post-immersion) for each group were shown as mean and SD and median and interquartile range (IQR); intergroup comparisons of these differences were performed using the Games-Howell *post hoc* test. All calculations were performed with SPSS version 26 (IBM Corp., Armonk, NY, US). The level of significance was set at p < 0.05.

## RESULTS

The pH measurement for Closeup® was neutral, while for Listerine® and Colgate® Plax it was acid in nature; the pH in Listerine® was 30.1% more acid than in Colgate® Plax (Table 1).

**Table 1:** pH of mouthwashes and microhardness of composite dental restorations before and after immersion in in each mouthwash

Mouthwash	pH	n	Pre-Immersion	Post-Immersion	p-value*
			Mean (SD)	Mean (SD)	
Closeup® Antibacterial Mouthwash Cool Breeze	7.02	10	50.45 (0.65)	51.40 (0.65)	0.015*
Colgate® Plax	5.62	10	50.66 (0.75)	50.35 (0.85)	0.301
Listerine® Teeth and Gum Defense	4.34	10	50.40 (0.79)	50.01 (1.68)	0.574

*: paired t-test.

Pre-immersion composite microhardness was very similar in the three mouthwash groups (range: 50.40-50.66).

We observed a reduction in microhardness in composite samples immersed in the Colgate® and Listerine® mouthwashes (-0.61% and -0.77%, respectively), although without statistical significance. The greatest reduction of microhardness was seen for the Listerine® mouthwash group. Conversely, a significant increase of composite microhardness was found for the Closeup® mouthwash group (+1.88%, p = 0.015) (Table 1).

The differences in microhardness pre- and post-immersion in each mouthwash were calculated and showed a great variability (Table 2). The increase in microhardness observed for Closeup® was significantly different from the decrease for Colgate®. Moreover, although we found a greater reduction in composite microhardness with Listerine® than with Colgate®, the difference was not statistically significant.

**Table 2:** Inter-group comparison of differences in microhardness of composite after immersion in each mouthwash

Mouthwash	Difference post- pre		Compared to	p-value (Games-Howell)
	Mean (SD)	Median (IQR)		
Closeup®Antibacterial Mouthwash Cool Breeze	0.95 (0.99)	0.72 (1.66)	Colgate® / Listerine®	0.021 / 0.206
Colgate® Plax	-0.31 (0.89)	-0.08 (1.67)	Listerine®	0.993
Listerine® Teeth and Gum Defense	-0.39 (2.11)	-0.70 (3.80)	-	-

## DISCUSSION

Many studies have reported negative effects of alcohol-containing mouthwashes on composite restorations^2^^,^^16^^-^^19^. George *et al.*^16^ and Kocchar *et al.*^17^, describe how the presence of alcohol in Listerine® decreases the hardness of dental restorative materials ^18^ ^19^.

Here, we show some reduction of microhardness even with alcohol-free mouthwashes, in line with previous studies reporting that alcohol-free mouthwashes may also affect the properties of composite restorations^20^. It is very important to be aware of the ingredients when using a mouthwash, as some may have negative effects on dental restorations.

The insignificant reduction of microhardness in the Colgate® Plax and Listerine® groups is in line with the results of a study carried out by Urbano et al.^21^ The reduction in microhardness observed with Listerine® is higher than in the other groups, which may be attributed to benzoic acid, one of its components.

Among the group of studied mouthwashes, Listerine® has the lowest pH; its acidic nature may lower composite microhardness^22^^,^^23^ and reduce oral pH, which affects the matrix of the polymer and reduce its microhardness^24^. Previous studies support these results^16^^,^^25^, while other studies indicate that the presence of sodium fluoride (a component in Listerine®) may reduce surface hardness^8^^,^^26^^,^^27^.

Our study shows an increase in microhardness in the Closeup® group, probably due to the presence of zinc in the mouthwash; the addition of zinc to the composite resin may enhance its mechanical properties^28^. Moreover, the Closeup® mouthwash has a neutral pH (7.02), and therefore there is no negative acid effect on microhardness^24^.

There are some limitations to our *in vitro* study. Firstly, the effect of the oral environment was not considered, including the presence of saliva, food, beverages, or the pH of the oral cavity. The small sample size may explain the high pre- and post-immersion heterogeneity and the inability to detect inter-group differences in some cases. Further studies should be developed with larger sample size and different types of mouthwashes and composite restorative materials for a comprehensive understanding of the effect of mouthwashes on the microhardness of such restorations.

The findings of this study suggest that alterations on the surface microhardness of restorative composites, either negative (with Colgate® and Listerine®) or positive (with Closeup®), are associated to the composition of the mouthwash. The adverse effect of mouthwashes on dental restorations seems to be caused by their acidic content and low pH (clearly seen with Listerine®); thus, it is advisable to limit the use of this type of mouthwashes to cases for which they are prescribed; furthermore, mouthwashes that contain acids or have low pH should not be prescribed.

## APPENDIX I

**Table:** Composition of mouthwashes

Mouthwash	Composition	Manufacturer
Closeup® Antibacterial Mouthwash Cool Breeze	Water, Sorbitol, PEG 40 Hydrogenated Castor Oil, Potassium Citrate, Glycine, Benzyl Alcohol, Phenoxyethanol, Sodium Lauryl Sulfate, Perfume, Zinc Sulfate, Sodium Saccharin, Sodium Fluoride, CI 42090, Eugenol, Limonene, Linalool.	Unilever,
	Available Fluoride: max 250 ppm.	Russia
Colgate® Plax	Water, Glycerine, Propylene Glycol, Sorbitol, Poloxamer 407, Flavour, Cetylpyridinium Chloride, Potassium Sorbate, Sodium Fluoride, Sodium Saccharine, Menthol	Colgate-Palmolive,
	Available Fluoride: 225ppm	Thailand
Listerine® Teeth and Gum Defence	Aqua, Sorbitol, Propylene Glycol, Sodium Lauryl Sulfate, Poloxamer 407, Benzoic Acid, Eucalyptol, Methyl Salicylate, Thymol, Sodium Saccharin, Sodium Fluoride, Sodium Benzoate, Menthol, Aroma, Benzyl Alcohol, Sucralose	Johnson & Johnson, Italy
	Available Fluoride: 220 ppm	
